# The role of artificial intelligence in sports training: opportunities, challenges and future applications for competitive swimming

**DOI:** 10.5114/biolsport.2026.152352

**Published:** 2025-09-16

**Authors:** Luca Puce, Piotr Żmijewski, Filippo Cotellessa, Cristina Schenone, Halil I. Ceylan, Nicola L. Bragazzi, Carlo Trompetto

**Affiliations:** 1 Department of Neuroscience, Rehabilitation, Ophthalmology, Genetics, Maternal and Child Health, University of Genoa, Genoa, Italy; 2 IRCCS Ospedale Policlinico San Martino, Genoa, Italy; 3 Jozef Pilsudski University of Physical Education in Warsaw, Poland; 4 Physical Education and Sports Teaching Department, Faculty of Sports Sciences, Ataturk University, Erzurum, Turkey

**Keywords:** Natural language processing, AI-driven chatbots, Periodisation phase, Sprinters, Distance swimmers

## Abstract

AI-based chatbots are increasingly used to design training programs, but their effectiveness for elite athletes is unclear. This study assessed ChatGPT-4’s ability to generate weekly training plans for elite swimmers and sprinters. Twenty-three coaches and thirty-six athletes rated the AI-generated plans using a 5-point Likert scale in three areas: weekly frequency, intensity adjustments, and training structure. Seven intensity zones were analyzed: A1 (endurance/recovery), A2 (extensive aerobic), B1 (intensive aerobic), B2 (aerobic-anaerobic transition), C1 (anaerobic threshold), C2 (anaerobic-lactate), and C3 (maximal sprint intensity). Coaches gave neutral-to-positive ratings (3.6 for distance swimmers, 3.7 for sprinters), while athletes were more critical (2.8 and 3.1, respectively). AI-generated plans performed well in low-intensity zones (A1) but had shortcomings in moderate-intensity (A2, B1–B2: long repetitions, excessive sets, insufficient recovery) and anaerobic zones (C1: excessive frequency for swimmers; C2–C3: insufficient frequency for sprinters). No significant differences emerged between plans for swimmers and sprinters (p=0.596), but A2, B1, and B2 showed greater discrepancies (p < 0.001). Rating reliability was moderate for coaches (ICC=0.609) and low for athletes (ICC=0.369). Older coaches and male athletes rated the plans lower, while those with national-level experience were more favorable. While 65% of coaches found the plans usable with minor modifications, only 27.8% of athletes agreed, 47.2% requested major changes, and 25% rejected them. ChatGPT-4 is useful for simple training plans but requires human supervision for complex periodization, particularly in high-intensity zones.

## INTRODUCTION

Artificial Intelligence (AI) encompasses a broad spectrum of technologies designed to execute tasks traditionally requiring human intelligence. Among these, natural language processing (NLP) models, such as AI-driven chatbots, are transforming how we interact with complex data [1, 2]. These chatbots illustrate AI’s potential to bridge the divide between complex information and practical applications, thereby making advanced technology accessible to a wider audience [3, 4].

AI chatbots, trained on extensive datasets from diverse sources, can generate human-like responses and interpret information across various domains. Their versatility enables support in fields such as medicine, where they assist in analyzing medical literature and offering general recommendations [5, 6], biology, where they synthesize complex research findings [7, 8], and nutrition and wellness, where they deliver user-friendly, science-based advice [9, 10].

One of the most prominent AI tools in this domain is ChatGPT, developed by OpenAI. Its latest iteration, ChatGPT-4, represents a significant advancement over its predecessor, ChatGPT-3.5, offering improved capabilities in generating accurate, coherent, and contextually appropriate responses [11]. Compared to other AI models such as Google Gemini (previously known as Google Bard) and Microsoft Copilot, ChatGPT-4.0 has demonstrated stronger performance in various specialized applications, reinforcing its potential as a training tool in sports science [9, 12, 13].

Recent studies have explored the capabilities of AI in generating exercise programs, highlighting both its strengths and limitations, particularly in specialized contexts. Xu et al. [14] compared ChatGPT-4.0 with an Intelligent Health Promotion System (IHPS) for prescribing exercise to hypertensive patients. The results showed that ChatGPT-4.0 outperformed the IHPS in generating accurate and comprehensive exercise plans. However, the AI tended to prioritize safety over effectiveness, particularly in complex medical scenarios, limiting its practical utility in some specific cases. Similarly, Dergaa et al. [15] found that ChatGPT-4.0-generated adapted exercise prescriptions lacked the precision required to address specific health conditions and personalized training needs, highlighting the irreplaceable role of expert human experience. In a related study, Zaleski et al. [16] examined the completeness, accuracy, and readability of exercise recommendations provided by ChatGPT-3.5. While the AI generally demonstrated high accuracy, significant gaps were identified in defining essential components such as frequency, intensity, duration, and type of physical activity-critical factors for effective and personalized exercise prescriptions.

Similar results have been found in more general contexts. Erol et al. [17] evaluated ChatGPT-3.5 responses for basic exercises in healthy individuals and found them to be accurate and satisfactory overall. However, human supervision remained essential for more complex and nuanced aspects of exercise programming.

Masagca et al. [18] evaluated ChatGPT-3.5-generated exercise programs for untrained university students. While these programs improved flexibility and muscular endurance, they were less effective than human-designed programs at improving cardiovascular endurance.

Concerning athletic populations, Washif et al. [19] analyzed 12-week resistance training programs generated by an AI chatbot for intermediate and advanced athletes. Although these programs adhered to scientific guidelines and periodization principles, expert intervention was required to fully align the training with specific goals. In this context, GPT-4 demonstrated greater adaptability and contextual understanding than GPT-3.5, but still required human oversight for proper adaptation. In support of these observations, Düking et al. [20] evaluated six-week training plans for runners generated by ChatGPT-3.5. Although not rated as optimal by expert coaches, the quality of these programs improved significantly with more detailed user input. Nevertheless, expert supervision remained critical to ensure adherence to evidence-based training principles. Similarly, Havers et al [21] examined the reproducibility and quality of resistance training programs focused on hypertrophy. Providing more specific input improved program quality, with GPT-4 outperforming Google Gemini in key aspects such as exercise selection, training volume, and advanced techniques. Despite these improvements, expert refinement was still required to ensure safety and alignment with best practices.

In summary, while AI-particularly GPT-4-has significant potential to generate exercise programs that are aligned with scientific guidelines, human oversight remains critical. The limitations of AI are particularly apparent in complex or highly personalized contexts, where human expertise is essential to ensure the accuracy, efficacy, and safety of exercise programs.

These challenges for AI can be even more pronounced in highly technical and competitive contexts, such as designing training programs for elite swimmers. In these cases, achieving maximum performance at key moments in the season requires a highly personalized and dynamic approach. It is essential to adjust key variables such as workload, recovery times and the physiological response of athletes in real time to optimize overall performance [22, 23].

An emblematic example is periodization, a structured approach divided into distinct phases (general, specialized, specific, and tapering), which is essential to optimize physiological capacities and ensure that athletes reach peak form at the time of competition [24, 25]. In particular, training programming for elite swimmers must account for the distinct physiological and technical demands of distance and sprint swimmers, which require specific adaptations in volume distribution, intensity, and recovery times. While distance swimmers need a strong aerobic base and prolonged endurance, sprinters must develop explosive power and high anaerobic output, making training strategies significantly different for each athlete category.

On average, during these phases, athletes swim up to 60 km per week at varying intensities, often training five to seven days per week [26–28]. On some training days, they may swim up to 14,000 meters, especially during double training sessions, a routine they maintain throughout their 10–15 year careers [27, 28].

Each phase of periodization has a specific physiological purpose. The general phase focuses on building a solid aerobic base, which is essential for sustaining the demands of subsequent training phases. As athletes move into the specific phase, training becomes more refined, with a progressive reduction in volume and an increasing emphasis on the development of aerobic power through threshold and ${\dot{\text{V}}\text{O}}_{2\max}$ exercises, as well as anaerobic power through lactate tolerance and peak lactate production training [29].

This phase is particularly crucial for race success, as it involves adapting training to replicate race conditions, making the precise balance between volume, intensity, and recovery a determining factor in performance. Even small errors in training load distribution or tapering strategies can significantly compromise an athlete’s peak race condition [24, 25, 30, 31].

Despite the growing integration of technology in elite sports, the role of AI in designing personalized training programs for elite swimmers remains virtually unexplored.

While existing research has evaluated AI-generated plans for a few athletic populations runners, no study has yet systematically analyzed whether AI can successfully replicate the nuanced, adaptive, and highly personalized approach required in elite swimming preparation.

To fill this gap, the present study aims to evaluate the reliability and accuracy of ChatGPT-4 in designing training programs for elite distance and sprint specialists during the specific periodization phase. By comparing the programs generated by the AI with the standards adopted by coaches and the judgment of expert athletes, the aim is to identify the strengths and limitations of the AI in elite swimming training.

Additionally, the study will assess whether these programs can be used in future research to evaluate their effectiveness in real-world conditions, by measuring technical improvements and performance outcomes over time.

We hypothesize that AI will be most effective in designing standardized, well-structured training sessions, such as those focused on aerobic base development, where training parameters are welldefined and supported by extensive literature.

However, for more complex training elements, such as aerobic power enhancement or anaerobic threshold development, human expertise is likely to remain essential.

Additionally, given the distinct physiological and technical demands of distance swimmers versus sprinters, it remains uncertain whether AI can effectively differentiate and optimize training plans for these two athlete profiles.

## MATERIALS AND METHODS

### Study Design

This study employed a mixed-methods approach, combining qualitative and quantitative techniques to evaluate the weekly training programs generated by ChatGPT-4 for elite swimmers during the “specific phase” of periodization.

These programs were assessed by expert coaches through faceto-face interviews conducted during national competitions in the 2024/2025 competitive season. The objective was to determine the degree of alignment with standard guidelines and the coaches’ field experience, highlighting strengths and weaknesses, providing suggestions for improvement, and assessing whether these programs can be used in future research to evaluate their effectiveness in real-world conditions by measuring technical improvements and performance outcomes over time.

Additionally, athletes were involved in the evaluation process. This allowed for the integration of a unique perspective, analyzing not only the theoretical quality of the programs but also their actual applicability and impact on athletic performance.

To enhance the analysis, both coaches and experienced athletes completed a comprehensive questionnaire. Coaches provided information on age, gender, and years of experience at national and international levels. Similarly, athletes reported their age, gender, and the number of years of competitive training. Additionally, all participants were required to self-report their prior experience with AIbased tools in sports training. They rated their familiarity and usage of AI-driven technologies, such as automated performance analysis systems, AI-powered training assistants, and machine learning-based injury prevention tools, on a five-point Likert scale (1 = no experience, 5 = extensive experience).

The study strictly followed established ethical guidelines, ensuring the confidentiality of participants and obtaining informed consent in accordance with the requirements of the local ethics committee. The study protocol was formally reviewed and approved by the local ethics committee of the University of Genoa, Genoa, Italy (protocol number 2025.03).

### Participants

The selected coaches met specific inclusion criteria, including holding a Level II certification from the Italian Swimming Federation, active participation in national and/or international competitions, and a minimum of five years of experience coaching elite athletes. Initially, 31 coaches were selected, but 8 declined to participate due to time constraints, despite expressing enthusiasm for the project. In total, 23 coaches participated in the study, including 9 women. The average age of the participants was 42.91 ± 7.83 years, ranging from 31 to 65 years. Their average national-level coaching experience was 17.87 ± 8.02 years (range: 7–43 years), while their international experience averaged 3.65 ± 2.84 years (range: 0–10 years). Their experience with AI-based tools in sports training reached an average Likert scale score of 2.0 ± 0.8.

For the athletes, 45 were initially recruited, but only 36 completed the evaluation process. The inclusion criteria required a minimum of 2 years of competitive training at the national or international level. The average age of the participating athletes was 17.8 ± 1.7 years, ranging from 16 to 23 years. The sample consisted of 22 men and 14 women. Their average competitive experience was 5.5 ± 1.6 years, with a range of 3–9 years. Their experience with AI-based tools in sports training reached an average Likert scale score of 2.1 ± 1.1.

### Prompt Design

The weekly training plan was structured according to the international training classification systems and includes three physiologically defined intensity zones: aerobic, power aerobic, and anaerobic [32]. The practical application of these zones has been optimized using parameters that have been validated over time in the assessment of swimming performance, including heart rate (HR) thresholds, blood lactate concentrations (mmol/L), and oxygen uptake kinetics (${\dot{\text{V}}\text{O}}_{2\max}$) [29, 31, 33].

The aerobic domain includes three subzones: low-intensity efforts for active recovery (A1), moderate-intensity efforts below the anaerobic threshold to develop aerobic capacity (A2), and threshold-intensity efforts aimed at improving performance at the anaerobic threshold (B1) [34]. The aerobic performance domain (B2) includes maximal sustained intensity efforts to stimulate peak oxygen uptake (${\dot{\text{V}}\text{O}}_{2\max}$) [35].

The anaerobic domain is designed to optimize lactate tolerance and power output through three distinct subzones: high-intensity efforts to maximize lactate production (C1), short bursts of maximal power in an anaerobic alactic state (C2), and explosive movements or all-out efforts to increase neuromuscular efficiency and anaerobic alactic capacity (C3) [36].

**FIG. 1:**
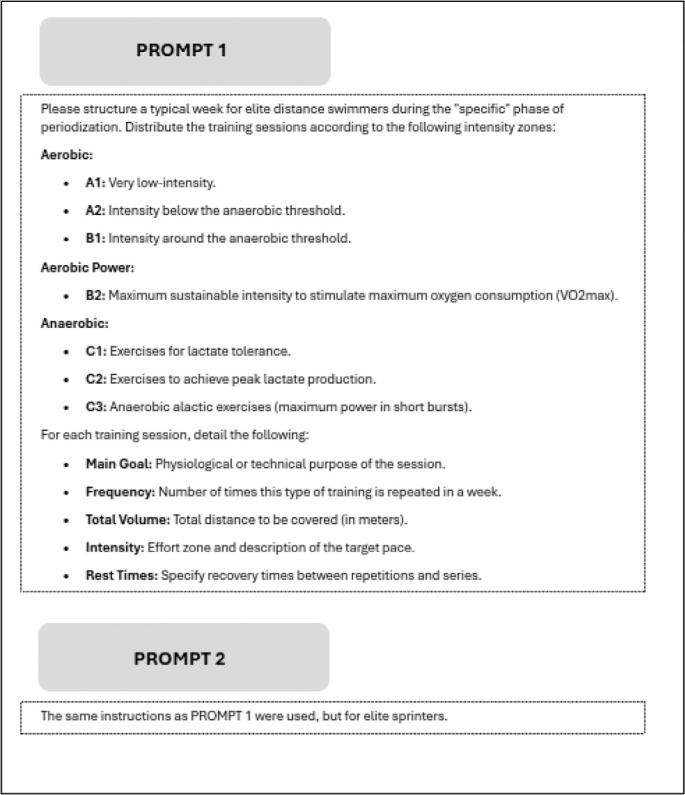
Instructional prompt provided for designing a weekly training program for elite swimmers during the “specific” phase of periodization. The prompt categorizes intensity zones into aerobic and anaerobic categories, specifying the main goals, frequency, total volume, intensity, and recovery times for each training session. Two versions are presented: one for distance swimmers (Prompt 1) and another for sprinters (Prompt 2).

Detailed parameters were defined for each intensity zone, specifying the specific purpose, weekly frequency, total volume, intensity and recovery periods for each session [33] (Figure 1). The classification codes (A1, A2, B1, B2, C1, C2, C3) correspond to those used by the Italian Swimming Federation [37]. Although different national federations use different classification codes, they generally correspond to the same physiological parameters [38].

### Response Evaluation

The response evaluation involved classifying each training intensity zone on a five-point Likert scale according to the fundamental criteria of training periodization and their direct impact on the adaptation of performance in elite swimmers [31].

The first criterion, Frequency of Zone-Specific Sessions, measured the number of weekly sessions targeting each intensity zone. The second criterion assessed the Correspondence Between Prescribed Intensity and Training Zone, evaluating the alignment between the prescribed intensity for each session and the programmed training zone, expressed as a percentage of the maximum heart rate (HR_max_). Finally, the third criterion examined the Exercise Structure for Each Session, considering both load density (the balance between exercise intensity and recovery periods) and load quantity (the total workload relative to session duration, repetitions, stimuli, and prescribed distances).

The Likert scale ranged from 1 to 5, where 1 corresponded to “Strongly Disagree” and 5 to “Strongly Agree” [39]. Alongside these ratings, detailed notes on errors and suggestions for improvement were provided for each training intensity zone assessed.

Additionally, an evaluation was conducted to determine whether these programs could be used in future research to assess their effectiveness in real-world conditions by measuring technical improvements and performance outcomes over time. A custom scale was utilized for this purpose: “Yes,” “Yes, but with slight modifications,” “Yes, but with significant modifications,” and “No.” This assessment aimed to provide insights into the practical applicability of the training programs and their potential for refinement in future studies.

### Statistical Analysis

Prior to analysis, the data were visualised and checked for outliers and normality of distribution. Descriptive statistics were used to summarise the data, presented as means and standard deviations.

Comparability and agreement between coaches and athletes was assessed using the intraclass correlation coefficient (ICC) based on a two-way random effects model for consistency (ICC (2,k)). This approach was chosen to assess the level of agreement between coaches and athletes who were representative of a larger population of similar raters. The analysis considered variability attributable to both the coaches or athletes and the factors being assessed, including type of plan (e.g. distance specialists and sprinters) and intensity zones. In addition, the model included the average of the coaches’ and athletes’ measurements to provide a more robust measure of agreement. A multivariable linear regression analysis was conducted to investigate the factors influencing coaches’ and athletes’ judgments of sprinters’ and distance swimmers’ training plans. The analysis assessed the potential impact of the independent variables, including coaches’ and athletes’ gender, age, years of experience, and experience with AI-based tools, on the dependent variable (training plan judgments).

Finally, the differences in ratings between the training plans of distance specialists and sprinters were analysed, focusing on the intensity zones within these plans. Non-parametric pairwise tests were used for analysis. The effect size was computed using the rank-biserial correlation coefficient. Absolute values ranging from 0.00 to 0.10 indicated a negligible effect, while those between 0.10 and 0.30 suggested a small effect. A moderate effect was observed when the coefficient fell within the range of 0.30 to 0.50, whereas values between 0.50 and 1.00 denoted a large effect.

All results are presented with corresponding p-values, with statistical significance set at p < 0.05. Where necessary, corrections for multiple comparisons were applied to ensure the robustness of the results.

## RESULTS

### Weekly Training Plan for distance specialists

**FIG. 2:**
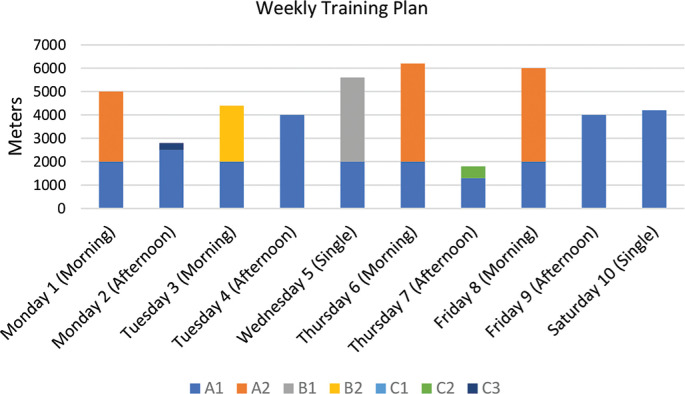
Weekly training plan for distance swimming specialists, showing the breakdown of swimming volume per session for each specific intensity zone (A1, A2, B1, B2, and C1).

The training week was divided into a total of ten sessions spread over six days. The sessions were divided into morning and afternoon sessions, with a single session on Wednesday and Saturday. Mondays, Tuesdays, Thursdays and Fridays were double sessions (morning and afternoon). The total training volume for the week was 62,100 m, divided into different intensity zones (Figure 2, Table 1).

**TABLE 1:** Detailed weekly training plan for elite distance swimmers, presenting the goals, training volumes (in meters), intensity zones (% of HR_max_), and session structures for each day.

Day/Session	Goal	Volume (m)	Intensity Zone (HR_max_)	Structure Summary
Monday 1 (Morning)	A1	1000	~60%	1,000 m warm-up
	A2	6000	~70–75%	4 × 1,500 m steady (1’ rest)
	A1	500	~60%	500 m easy

Monday 2 (Afternoon)	A1	1000	~60%	1,000 m warm-up
	C1	1600	~90–95%	8 × 200 m (150 m fast + 50 m easy, 1’30’’ rest)
	A1	1000	~60%	1,400 m cool-down

Tuesday 3 (Morning)	A1	1000	~60%	1,000 m warm-up;
	B2	3600	~90–95%	12 × 300 m fast (1’ rest)
	A1	1400	~60%	1,400 m cool-down

Tuesday 4 (Afternoon)	A1	1000	~60%	1,000 m warm-up
	A1	3000	~60%	3 × 1,000 m continuous (2’ rest)
	A1	1000	~60%	1,000 m cool-down

Wednesday 5 (Single)	A1	1000	~60%	1,000 m warm-up
	B1	4800	~85%	6 × 800 m at threshold pace (1’30’’ rest)
	A1	1200	~60%	1,200 m cool-down

Thursday 6 (Morning)	A1	1000	~60%	1,000 m warm-up
	A2	4800	~70–75%	4 × 1,200 m steady (1’ rest)
	A1	1200	~60%	1,200 m cool-down

Thursday 7 (Afternoon)	A1	1000	~60%	1,000 m warm-up
	C1	1500	~90–95%	10 × 150 m (100 m fast + 50 m easy, 1’ rest)
	A1	1500	~60%	1,500 m cool-down

Friday 8 (Morning)	A1	1000	~60%	1,000 m warm-up
	A2	6000	~70–75%	5 × 1,200 m (1’ rest)
	A1	1000	~60%	1,000 m cool-down

Friday 9 (Afternoon)	A1	1000	~60%	1,000 m warm-up
	A1	3000	~60%	3 × 1,000 m continuous (2’ rest)
	A1	1000	~60%	1,000 m cool-down

Saturday 10 (Single)	A1	8000	~60–65%	2 × 4,000 m continuous (2’ rest)
	A1	1000	~60%	1,000 m cool-down

Intensity zone A1 (~60% HR_max_) contributed to a total of 33,800 m and was the main load in all sessions. This zone was used for warm-up, fatigue and continuous low-intensity work, with volumes ranging from a minimum of 1500 m (Monday morning) to a maximum of 9000 m (Saturday, single session).

Zone A2 (~70–75% HR_max_) reached a total volume of 16,800 m, the second largest contribution of the week. Sessions in this zone consisted of long, steady efforts, including low intensity repetitions over longer distances. The weekly sessions included the 4 × 1,500 m on Monday morning, the 4 × 1,200 m on Thursday morning and the 5 × 1,200 m on Friday morning.

Zone B1 (~85% HR_max_) totaled 4,800 m, all completed during the Wednesday session, which featured 6 × 800 m. Zone B2 (~90–95% HR_max_) contributed 3,600 m, exclusively from Tuesday morning’s session of 12 × 300 m.

Zone C1 (~90–95% HR_max_) logged a volume of 3,100 m, distributed between Monday afternoon (1,600 m from 8 × 200 m) and Thursday afternoon (1,500 m from 10 × 150 m).

No volume was recorded in zones C2 and C3 during the week.

### Evaluation for Distance Specialists

Coaches assigned an overall average score of 3.6 ± 1.2 for all intensity zones. The mean values for individual intensity zones, based on the weekly frequency of zone-specific sessions, intensity alignment, and the structure of exercises in each session, were: A1 (3.7 ± 0.1), A2 (3.6 ± 0.3), B1 (3.7 ± 0.1), B2 (3.6 ± 0.1), and C1 (3.5 ± 0.1).

Athletes rated with an overall average score of 2.8 ± 1.2. The mean values for individual intensity zones were: A1 (2.7 ± 1.2), A2 (2.6 ± 1.2), B1 (2.7 ± 1.2), B2 (2.8 ± 1.4), and C1 (3.3 ± 0.9).

Regarding the weekly frequency of zone-specific sessions, coaches assigned the highest scores to zones B1 (4.0 ± 0.6) and B2 (4.7 ± 0.6). Zones A1 (3.7 ± 0.6) and A2 (3.8 ± 0.9) received moderate scores, while zone C1 (2.6 ± 0.7) recorded a relatively low score. Athletes rated zones B2 (3.6 ± 0.9) and B1 (3.2 ± 0.6) with the highest scores. Zones A1 (2.6 ± 0.8) and A2 (2.8 ± 1.0) received moderate scores, while zone C1 (2.6 ± 0.0) recorded a relatively low score.

Regarding intensity zone alignment, coaches assigned very high scores to zones A1 (4.8 ± 0.5) and A2 (4.9 ± 0.3), as well as to zones B1 (4.9 ± 0.3) and B2 (4.7 ± 0.6). Zone C1 (4.3 ± 0.6) demonstrated good alignment, though with slightly lower values compared to the other zones. Athletes rated zones A1 (3.9 ± 0.7) and A2 (3.7 ± 0.7) with high scores, followed by B1 (3.6 ± 0.6) and B2 (3.6 ± 0.7). Zone C1 (3.2 ± 0.8) achieved good alignment but had lower scores compared to the other zones.

Regarding the structure of exercises in each session, coaches assigned relatively low scores to zones A1 (2.7 ± 0.8) and A2 (2.3 ± 0.6), with similar results for zone B1 (2.1 ± 0.5). Zone B2 (1.3 ± 0.4) obtained the lowest score, indicating significant room for improvement. In contrast, zone C1 (3.6 ± 0.6) recorded the highest score, demonstrating superior structuring of exercises compared to the other zones. Athletes expressed similar ratings, with low scores for zones A1 (1.7 ± 0.7), A2 (1.4 ± 0.6), B1 (1.3 ± 0.6), and B2 (1.0 ± 0.0), while zone C1 (3.2 ± 0.8) obtained the highest score.

### Weekly Training Plan for sprinters

**FIG. 3:**
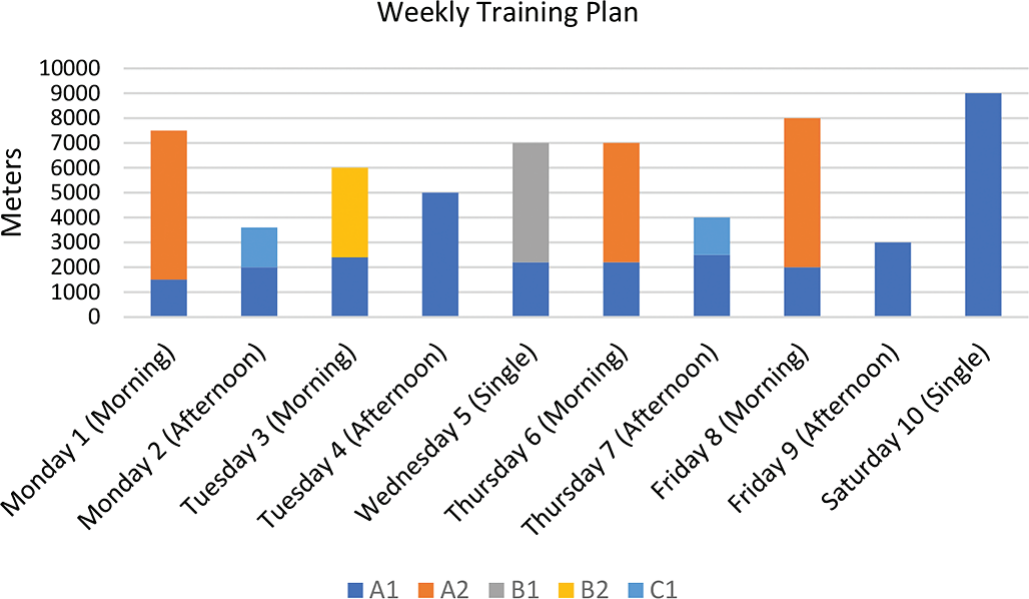
Weekly training plan for sprinters, showing the breakdown of swimming volume per session for each specific intensity zone (A1, A2, B1, B2, C2 and C3).

This week’s training consisted of ten sessions spread over six days, with double sessions on Monday, Tuesday, Thursday and Friday and single sessions on Wednesday and Saturday. The total volume recorded was 45,800 m, divided between the different intensity zones (Figure 3, Table 2).

**TABLE 2:** Detailed weekly training plan for sprinters, presenting the goals, training volumes (in meters), intensity zones (% of HR_max_), and session structures for each day.

Day/Session	Goal	Volume (m)	Intensity Zone (HR_max_)	Structure Summary
Monday 1 (Morning)	A1	1,000	~60%	1,000 m warm-up
	A2	3,000	~70–75%	4 × 750 m steady swim (30” rest)
	A1	1,000	~60%	1,000 m cool-down

Monday 2 (Afternoon)	A1	1,000	~60%	1000 m warm-up
	C3	300	~95–100%	12 × 25 m max effort sprints (3’ rest)
	A1	1,500	~60%	1500 m cool-down

Tuesday 3 (Morning)	A1	1,000	~60%	1,000 m warm-up
	B2	2,400	~90–95%	8 × 300 m @ ${\dot{\text{V}}\text{O}}_{2\max}$ pace (2’ rest);
	A1	1,000	~60%	1,000 m cool-down

Tuesday 4 (Afternoon)	A1	800	~60%	800 m warm-up
	A1	2,400	~60%	3 × 800 m continuous swim (30” rest)
	A1	800	~60%	800 m cool-down

Wednesday 5 (Single)	A1	1,000	~60%	1,000 m warm-up
	B1	3,600	~85%	6 × 600 m (1’ rest)
	A1	1,000	~60%	1,000 m cool-down

Thursday 6 (Morning)	A1	1,000	~60%	1,000 m warm-up
	A2	4,200	~70–75%	6 × 700 m steady swim (1’ rest)
	A1	1,000	~60%	1,000 m cool-down

Thursday 7 (Afternoon)	A1	500	~60%	500 m warm-up
	C2	500	~90–95%	6 × 50 m all-out (3’ rest); 8 × 25 m sprints from blocks (2’ rest)
	A1	800	~60%	800 m cool-down

Friday 8 (Morning)	A1	1000	~60%	1,000 m warm-up
	A2	4,000	~70–75%	5 × 800 m @ moderate intensity (45” rest)
	A1	1000	~60%	1,000 m cool-down

Friday 9 (Afternoon)	A1	800	~60%	800 m warm-up
	A1	2,400	~60%	3 × 800 m @ easy pace (30” rest)
	A1	800	~60%	800 m cool-down

Saturday 10 (Single)	A1	1000	~60%	1,000 m warm-up
	A1	4000	~60–65%	2 × 2,000 m continuous swim (2’ rest)
	A1	1000	~60%	1,000 m cool-down

The A1 intensity zone (~60% HR_max_) was the main component of the training, with a total contribution of 27,800 m. This zone was present in all sessions and was used for warm-up, fatigue and continuous low-intensity work. The most significant examples are the single session on Saturday, which included a continuous long swim of 2 × 2000 m, and the afternoon sessions on Tuesday and Friday, which included continuous runs of 3 × 800 m for both, with short recoveries between intervals.

Zone A2 (~70–75% HR_max_) totalled 11,200 m and contributed significantly to the weekly load. It was used for prolonged and continuous work, such as the 4 × 750 m continuous swim on Monday morning, the 6 × 700 m on Thursday morning and the 5 × 800 m on Friday morning.

Zone B1 (~85% HR_max_) completed a total of 3,600 m, concentrated on Wednesday’s session, which included 6 × 600 m. Zone B2 (~90–95% HR_max_) recorded a volume of 2,400 m, distributed over Tuesday morning’s session of intense work of 8 × 300 m.

The high intensity zones C1 (~95–100% HR_max_) and C2 (~90–95% HR_max_) were used in limited volumes, reflecting their purpose of speed and anaerobic power training. Zone C3 was present on Monday afternoon for a total of 300 m, which included 12 × 25 m maximum sprints. Zone C2 was present on Thursday afternoon, with a total of 500 m split between explosive work, including 6 × 50 m all-out and 8 × 25 m sprints from the blocks.

### Evaluation for Sprinters

Coaches assigned an overall average score of 3.7 ± 1.4 for all intensity zones. The average scores for individual intensity zones were: A1 (4.3 ± 0.5), A2 (3.5 ± 1.6), B1 (3.4 ± 1.8), B2 (3.4 ± 2.0), C2 (3.8 ± 1.4), and C3 (3.7 ± 1.8). Athletes assigned an overall average score of 3.1 ± 1.4, with the following values for individual intensity zones: A1 (3.1 ± 1.0), A2 (2.6 ± 1.4), B1 (2.6 ± 1.3), B2 (2.7 ± 1.4), C2 (3.6 ± 1.2), and C3 (3.7 ± 1.6).

For the weekly frequency of zone-specific sessions, coaches attributed the highest score to zone B2 (4.4 ± 0.7), followed by zones A2 (4.0 ± 0.7) and B1 (4.0 ± 0.7). Zone A1 recorded a score of 3.7 ± 0.6, while zones C2 (2.3 ± 0.7) and C3 (1.6 ± 0.6) obtained lower scores. Athletes gave the highest score to zone B2 (3.3 ± 0.8), followed by zone A2 (3.0 ± 1.1) and zone B1 (2.9 ± 0.9). Zone A1 received a score of 2.5 ± 0.9, while zones C2 (2.2 ± 0.7) and C3 (1.6 ± 0.6) recorded the lowest values.

Regarding intensity zone alignment, coaches assigned the highest score to zone C2 (5.0 ± 0.2), followed by A2 (4.9 ± 0.3), A1 (4.7 ± 0.5), B1 (4.8 ± 0.5), and B2 (4.8 ± 0.4). Zone C3 recorded a score of 4.9 ± 0.5. Athletes rated zone C2 (4.4 ± 0.7) the highest, followed by zones A2 (3.8 ± 0.7), A1 (3.6 ± 0.7), B1 (3.9 ± 0.6), and B2 (3.8 ± 0.9), with C3 at 4.8 ± 0.5.

Regarding the structure of exercises in each session, coaches assigned the highest score to zone C3 (4.5 ± 0.6), followed by A1 (4.4 ± 0.7) and C2 (4.2 ± 0.6). Lower ratings were given to A2 (1.7 ± 0.7), B1 (1.3 ± 0.5), and B2 (1.1 ± 0.3). Athletes also ranked zone C3 the highest (4.5 ± 0.6), followed by C2 (4.2 ± 0.6) and A1 (3.7 ± 0.9), while zones A2 (1.4 ± 0.6), B1 (1.2 ± 0.4), and B2 (1.0 ± 0.2) received the lowest scores.

### Evaluation of Feasibility for Real-World Application

Fifteen out of 23 (65%) of the coaches are in favor of a future practical application, albeit with slight modifications, while eight (34.8%) would support it only with significant modifications. Regarding the athletes, ten out of 36 (27.8%) are in favor with slight modifications, seventeen (47.2%) are in favor but with significant modifications, while nine (25%) are absolutely against it.

### Comparison of the plans

**FIG. 4:**
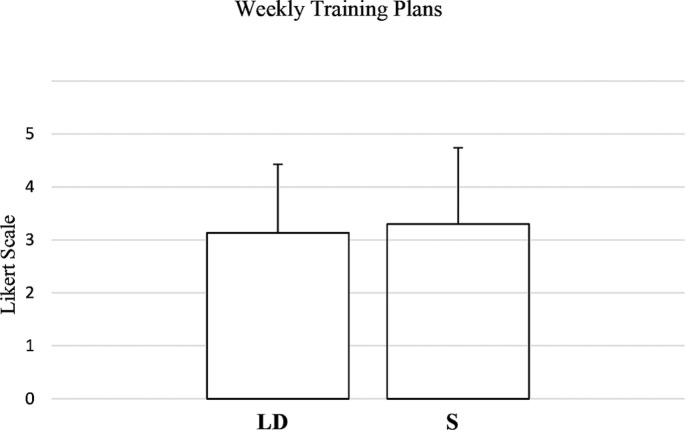
Coach and athlete evaluation of AI-generated weekly training plan for long distance (LD) and sprint athletes (S).

Overall, the evaluation of the training plan did not differ significantly between long-distance swimmers and sprinters (W = 101693.0, p = 0.596, RBC = -0.0238, negligible effect) (Figure 4).

**FIG. 5:**
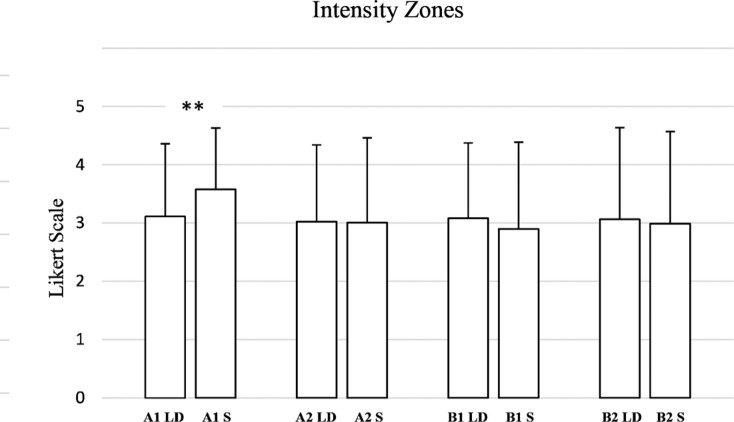
Evaluation by coaches and athletes of the AI-generated weekly training plan for long distance (LD) and sprint athletes (S), broken down into intensity zones. (A1: Very low-intensity; A2: Intensity below the anaerobic threshold; B1: Intensity around the anaerobic threshold; B2: Maximum sustainable intensity to stimulate maximum oxygen consumption). ** → p ≤ 0.01.

Concerning intensity zones (Figure 5), for A1 intensity, a statistically significant difference was observed (W = 3738.0, p = 0.002, RBC = -0.2938), indicating a small-to-moderate effect size. However, for A2 intensity, no significant difference was found (W = 5372.0, p = 0.874, RBC = 0.0150), indicating a negligible effect. In the B zones, differences were not statistically significant (W = 5719.0, p = 0.184, RBC = 0.1266, small effect). Similarly, B2 intensity yielded a Wilcoxon statistic of 5337.0 (p = 0.593, RBC = 0.0512, negligible effect).

**FIG. 6:**
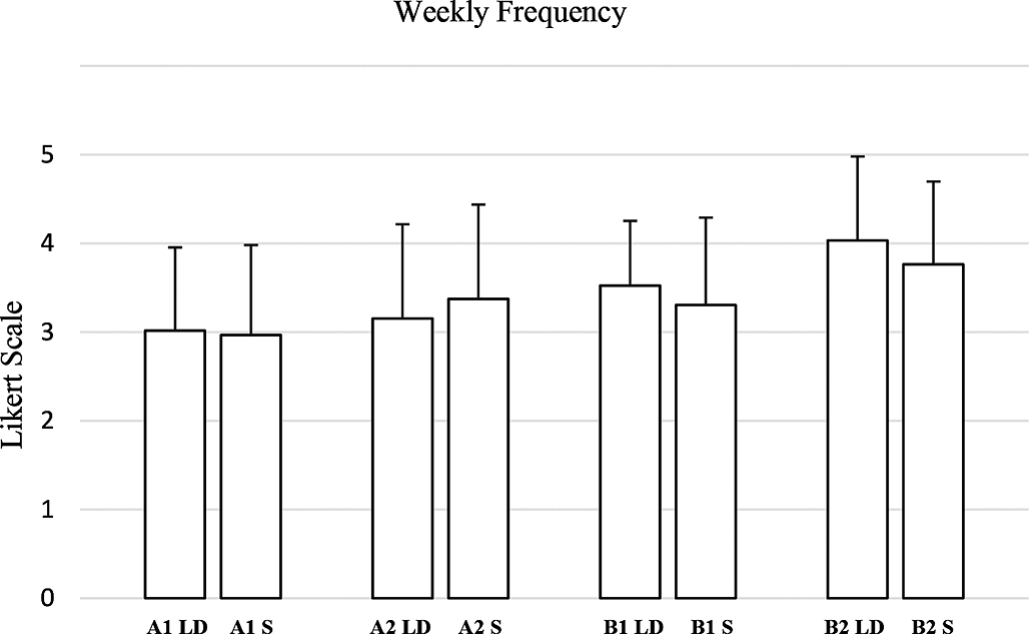
Evaluation by coaches and athletes of the weekly training plan generated by the AI for long-distance (LD) and sprint (S) athletes, divided into intensity zones in relation to “weekly frequency” criteria. (A1: Very low-intensity; A2: Intensity below the anaerobic threshold; B1: Intensity around the anaerobic threshold; B2: Maximum sustainable intensity to stimulate maximum oxygen consumption).

Regarding weekly frequency (Figure 6), no significant differences were observed in most comparisons. For A1 weekly frequency, the test yielded a Wilcoxon statistic of 286.0 (p = 0.664, RBC = 0.0833, negligible effect). Similarly, A2 weekly frequency showed a Wilcoxon statistic of 201.0 (p = 0.142, RBC = -0.2834, small effect). In contrast, B1 weekly frequency (W = 279.0, p = 0.069, RBC = 0.3744) and B2 weekly frequency (W = 282.0, p = 0.016, RBC = 0.4921) exhibited moderate to large effect sizes.

**FIG. 7:**
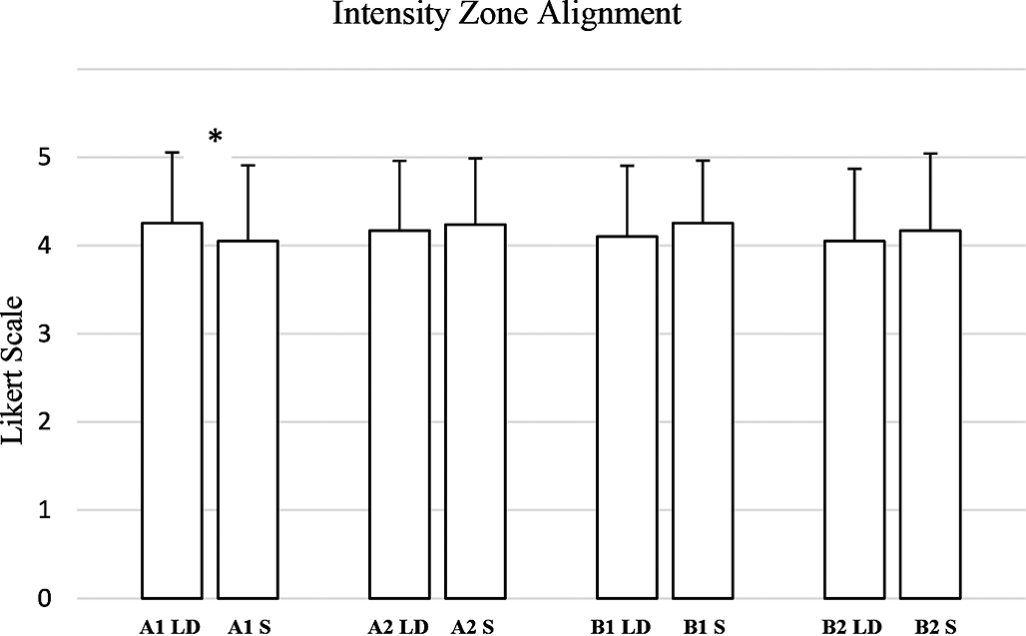
Evaluation by coaches and athletes of the weekly training plan generated by the AI for long-distance (LD) and sprint (S) athletes, divided into intensity zones in relation to “intensity zone alignment” criteria. (A1: Very low-intensity; A2: Intensity below the anaerobic threshold; B1: Intensity around the anaerobic threshold; B2: Maximum sustainable intensity to stimulate maximum oxygen consumption). * → p ≤ 0.05.

For intensity alignment (Figure 7), A1 intensity alignment showed a significant difference (W = 199.0, p = 0.042, RBC = 0.4420), indicating a moderate to large effect. However, A2 intensity alignment (W = 77.5, p = 0.461, RBC = −0.1842), B1 intensity alignment (W = 88.5, p = 0.100, RBC = −0.3587), and B2 intensity alignment (W = 55.0, p = 0.299, RBC = −0.2810) did not reach statistical significance, with effect sizes ranging from small to moderate.

**FIG. 8:**
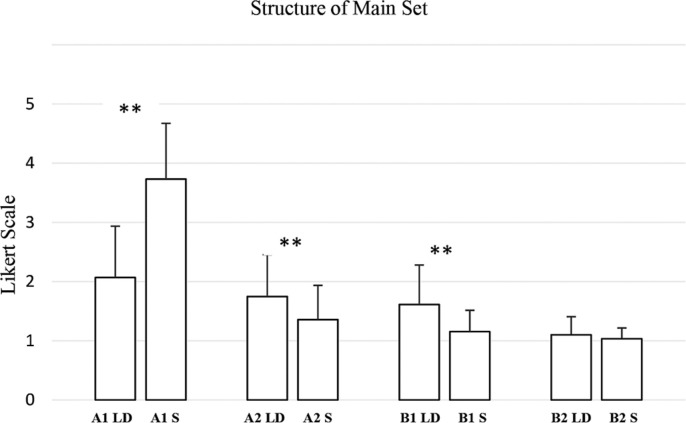
Evaluation by coaches and athletes of the weekly training plan generated by the AI for long-distance (LD) and sprint (S) athletes, divided into intensity zones in relation to “structure of main set” criteria. (A1: Very low-intensity; A2: Intensity below the anaerobic threshold; B1: Intensity around the anaerobic threshold; B2: Maximum sustainable intensity to stimulate maximum oxygen consumption). ** → p ≤ 0.01.

In contrast, the structure of the main set revealed the most pronounced differences (Figure 8). A1 structure of the main set (W = 11.0, p < 0.001, RBC = −0.9820), A2 structure of the main set (W = 301.0, p < 0.001, RBC = 0.8523), B1 structure of the main set (W = 300.0, p < 0.001, RBC = 1.0000), and B2 structure of the main set (W = 10.0, p = 0.072, RBC = 1.0000) all exhibited very large effect sizes.

Coaches’ agreement was moderate-to-good (ICC = 0.609 [95%CI 0.336–0.807], F = 2.559, p < 0.001). In the linear regression model (R^2^ = 0.574), age (β = −0.0806, p = 0.008) was a significant predictor, with increasing age associated with lower ratings. National experience (β = 0.0657, p = 0.016) was positively correlated with ratings, with more experienced coaches at the national level providing higher scores. Gender (p = 0.495), international experience (p = 0.531), and AI experience (p = 0.390) did not have a significant impact.

Finally, athletes’ agreement was low-to-moderate (ICC = 0.369 [95%CI 0.028–0.632], F = 1.584, p = 0.037). In the linear regression model (R^2^ = 0.159), only gender was a significant predictor, with male athletes (β = − 0.1493, p = 0.026) rating lower than female athletes. Age (p = 0.665), experience (p = 0.773), and AI expertise (p = 0.549) had no impact.

## DISCUSSION

This preliminary study investigated the potential of ChatGPT-4 to create weekly training plans tailored for elite swimmers during the “specific” phase of periodization, a critical phase focused on refining sport-specific skills and conditioning. The aim was to evaluate the accuracy and practicality of these AI-generated plans, laying the groundwork for future research to assess their real-world impact on technical and performance outcomes.

Feedback from coaches was generally cautious but optimistic. On a scale of perceived effectiveness, coaches gave a mean score of 3.6 for distance swimmers and 3.7 for sprinters. These results indicated a consensus that the plans fell between ‘neutral’ and ‘fairly effective,’ with slightly stronger agreement for sprinters. Athletes, however, expressed greater reservations and gave lower mean scores: 2.8 ± 1.2 for distance swimmers and 3.1 ± 1.4 for sprinters. Overall, considering the average scores of both coaches and athletes, there were no significant differences between the plans developed for distance swimmers and sprinters. However, this does not mean that the programmes are equally effective, as the qualitative analysis highlighted fundamental differences in training needs, such as the frequency of high intensity sessions for sprinters and the distribution of aerobic workload for distance swimmers, which will be discussed later.

Regarding the potential use of the programmes in future research to assess their effectiveness in real-life conditions, 65% of coaches said they would be willing to adopt the programmes with minor modifications, while 34.8% supported their use only after major modifications. Athletes, on the other hand, were more hesitant: only 27.8% agreed to use the programmes with minor modifications, while almost half (47.2%) required major modifications and 25% rejected their applicability altogether.

There could be several plausible reasons why athletes were more critical in their evaluations and more reluctant to accept the programmes than coaches. Having experienced the physical and mental demands of training first-hand, athletes are naturally more sensitive to potential imbalances: a programme that is perceived as inappropriate, insufficiently stimulating, or too demanding can lead to dissatisfaction [39, 40]. In addition, their expectations of innovation, effectiveness, and short-term results are often higher. If these expectations are not met, athletes are more likely to perceive the programme negatively [40].

These findings offer additional insight into the factors influencing the perceived effectiveness of AI-generated training programmes. Coaches’ moderate-to-good agreement suggests a relatively consistent evaluation among them, yet potential biases in their assessments should be considered. For instance, age emerged as a significant predictor, with older coaches tending to rate the plans lower—possibly reflecting a more conservative attitude toward technological innovations or stronger adherence to traditional training philosophies [41]. Conversely, national-level experience was positively correlated with higher ratings, indicating that more experienced coaches at higher competitive levels may recognize the potential value of innovative approaches like AI-generated plans.

Interestingly, factors such as gender, international experience, and AI experience did not show a significant impact, suggesting that professional exposure and personal coaching ideology, rather than technological familiarity, were the primary drivers of their assessments.

For athletes, the low-to-moderate agreement highlights greater variability in their perceptions, reinforcing the earlier observation of their heightened sensitivity to training demands. Gender was the only significant predictor in their evaluations, with male athletes providing lower ratings than female athletes. This difference may reflect variations in training expectations or responses to programme design across genders [42]. Other factors, such as age, experience, and AI expertise, did not significantly affect their evaluations, suggesting that personal perceptions and subjective experiences may play a more prominent role than background factors in shaping athletes’ judgments.

From the detailed analysis of the training plans Zone A1, which focuses on low-intensity aerobic training, proved to be the most effective for both distance swimmers and sprinters, achieving high scores on all evaluation criteria. This effect was even more statistically significant for sprinters, suggesting that AI excels in the design of standardised exercises based on common parameters that are predominantly adopted by less experienced swimmers.

Conversely, Zone A2 faced more significant criticism compared to Zone A1, particularly regarding the structure of the exercises. The Likert scale scores range from 1.7 for long distance specialists to 1.4 for sprinters, showing a significant difference. Both values correspond to a judgement of ‘disagree’. Some of the proposed sets were excessively long, such as 4,000 m with 800 m repetitions for sprinters and 6,000 m with 1,200 m repetitions for distance swimmers. Coaches recommended that main sets in Zone A2 range between 2,000 and 3,500 m, with shorter repetitions. For sprinters, an effective example might include *2 × (3 × 100 m + 2 × 200 m + 1 × 300 m)* with 15–30 seconds of recovery. For distance swimmers, a suitable structure could be *2 × (400 m + 300 m + 200 m + 200 m + 300 m + 400 m)* with 15–20 seconds of recovery.

Zones B1 (anaerobic threshold) and B2 (${\dot{\text{V}}\text{O}}_{2\max}$) also received negative evaluations regarding exercise structure, with significant discrepancies among examiners on aspects such as the number of sets, repetitions, and recovery intervals for both athlete groups. However, the proposed frequency and intensity for these zones were generally considered appropriate.

In Zone B1, ChatGPT-4 suggested sets such as *6 × 600 m with 1-minute rest* for sprinters (scored 1.2 “strongly disagree”) and *6 × 800 m with 1 minute 30 seconds rest* for distance swimmers (scored 1.6 “disagree”). Coaches justified this disagreement, recommending main sets between 1,500 and 2,000 m, with repetitions of 100–200 m to better align with training objectives. For instance, sprinters might benefit from a set of *10 × 150 m with 20 seconds rest*, while distance swimmers could perform *8 × 200 m with 30 seconds rest*.

In Zone B2, main sets should typically range between 1,200 and 1,600 m for both sprinters and distance swimmers, with repetitions of 200–400 m broken into shorter segments. Effective examples include *4 sets of 4 × 100 m* or *6 sets of 4 × 50 m*, incorporating short recoveries between repetitions and appropriate rest between sets. In contrast, ChatGPT-4 proposed continuous sets such as *8 × 300 m with 2 minutes rest* for sprinters (scored 1.0 “strongly disagree”) and *12 × 300 m at a fast pace with 1-minute rest* for distance swimmers (scored 1.1 “strongly disagree”).

Zone C1 was included only in the program for distance athletes, achieving an average score between “neutral” and “agree” (3.5 for distance swimmers and 3.3 for sprinters on the Likert scale). The most critical issue identified was the proposed frequency (scored 2.6 for both groups of athletes), as two weekly sessions in this zone were deemed excessive. Experts suggested that a single session would better align with physiological needs, as these workouts involve maximal or near-maximal intensity, pushing the body to the limits of its anaerobic capacity. For example, a typical session of *10 × 100 m sprints* with 1-minute recovery was highlighted by coaches as an effective yet demanding workout that necessitates careful scheduling. This high level of exertion requires longer recovery periods to prevent overtraining [43], reduce the risk of chronic fatigue, injury, and dissatisfaction, and avoid compromising the quality of training in other zones [27, 44, 45].

In the high-intensity zones (C2 and C3), not included in the program for distance athletes, sprinters rated the structure and intensity very highly. However, the frequency of sessions was rated negatively. In particular, for Zone C3 (scored 1.6 for both groups of athletes), which focuses on alactacid anaerobic metabolism, it is strongly recommended to increase the frequency of sessions significantly. Currently, only one session is offered, but coaches suggest that C3 sessions should be included almost daily as an activation phase before the main set, potentially replacing some A1 sessions. For example, short sprints of 15 m to a maximum of 25 m, starting from a stationary or block position, with up to 2 minutes of recovery between repetitions were recommended as an optimal way to develop explosive power and neuromuscular efficiency without inducing significant fatigue.

These results suggest that while ChatGPT-4 can generate highquality standardised training plans, such as low-intensity aerobic programmes, its limitations become apparent in more complex and highly specific contexts [24, 25]. Even for moderate-intensity aerobic training, the AI struggles to structure sets, despite adhering to appropriate physiological parameters and weekly frequency. The challenges are even more pronounced in threshold and ${\dot{\text{V}}\text{O}}_{2\max}$ training, particularly with regard to set construction. For pure anaerobic training, the main difficulty is in managing the weekly frequency: endurance athletes tend to overdo it, while sprinters underdo it.

As a result, human intervention remains essential to refine training structures and tailor programmes to the specific needs of athletes, especially at critical stages of periodisation. These difficulties are likely due to the fact that the areas where AI struggles most are those that are less documented in the literature, making it difficult to generate standardised, data-driven recommendations. AI models primarily process information from online sources, learning patterns and correlations from existing data [1, 2]. However, when specific literature on a given topic is scarce or non-existent, AI can produce inaccurate or suboptimal recommendations because it lacks a solid foundation on which to base its predictions [2]. In fact, there are few, if any, detailed guidelines on how to structure specific training series in terms of repetitions, sets and distances at different stages of preparation for threshold or anaerobic training. While general principles of periodisation exist, they often provide only broad guidelines, leaving coaches responsible for applying and adapting them based on their own experience, individual athlete responses and data collected in the field.

Comparison with the study by Erol and Arıkan [17], which analysed ChatGPT-3.5 in the design of core stability exercises, highlights significant similarities with our study, despite the use of a less advanced version than ChatGPT-4. In their study, the AI-generated responses were evaluated by a group of nine physiotherapists and rated as satisfactory but incomplete, with an average score of 3.93 out of 5. This rating reflects a lack of accuracy in certain details and highlights the inability of the model to monitor individual responses, adapt to the physical abilities of the subjects and consider their overall health status.

The study by Washif et al. [19] provides additional insight by investigating the use of ChatGPT in its 3.5 and 4.0 versions for the development of 12-week resistance training programmes. The results show that both models generated similar programmes in terms of progression, set structure and recovery timing. However, GPT-4 demonstrated a greater ability to personalise programmes, distinguishing between intermediate and advanced levels and including details such as active recovery and more sophisticated load progression, which is consistent with the findings of our study. However, the study highlights that AI-generated programmes still require expert customisation to achieve full effectiveness. While GPT-4 demonstrated a better understanding of training principles, it still has gaps, including the omission of innovative training methods and the inability to adapt to real-time feedback, which limits its usefulness in professional contexts. The authors conclude that ChatGPT can serve as a complementary tool for initial programme design but cannot replace human supervision.

The study by Zaleski et al. [16] further expands on AI-generated exercise recommendations, evaluating their comprehensiveness, accuracy, and readability. While the AI performed well in terms of factual accuracy (90.7%), it struggled with comprehensiveness (41.2%), indicating significant gaps in crucial areas such as exercise progression, volume, and specific medical clearance requirements. Furthermore, the study highlights that ChatGPT’s recommendations are written at a college-level reading difficulty, which could limit accessibility for general users. Notably, AI-generated outputs exhibited an overly cautious approach, often recommending unnecessary medical clearance before exercising, a finding that aligns with our study’s observation of AI’s conservative tendencies.

The comparison with the study by Masagca [18] highlights a recurring theme that is consistent with our study and the others cited: AI-generated programmes, while valid in terms of general structure, are less effective than those developed by human experts. Specifically, the AI-generated exercise programmes showed improvements in areas such as flexibility and muscular endurance but were inferior in cardiovascular endurance and overall coherence.

The study by Dergaa et al. [15] provides a different perspective, showing that GPT-4 tends to prioritise safety over effectiveness, producing programmes that emphasise conservative approaches rather than pushing for maximum performance. This finding is consistent with our study, in which numerous light aerobic sessions (zone A1) were proposed that coaches considered less useful than more challenging sets in zone C3. While this focus on caution is beneficial in general contexts, it is a significant limitation for competitive athletes who require highly specific programming to maximise their physical potential.

Finally, the study by Xu et al. [14] highlights further limitations of AI, underlining the difficulty of the model dynamically adapting to specific variations, thus confirming the need for human intervention to ensure programme customisation and effectiveness.

### Strengths and Limitations

One of the main strengths of this study is the participation of coaches and athletes trained exclusively within the Italian system, with only brief international experience for some coaches. This ensures a homogeneous educational and professional background. However, the results may reflect cultural and methodological nuances specific to the Italian coaching context, which may limit their generalisability to other systems.

Additionally, the study focused exclusively on ChatGPT-4 as the chatbot model for analysis and application. While this choice is justified by its status as one of the most advanced conversational AI models currently available, the exclusion of other chatbots (such as those from alternative platforms or developers) prevents a broader comparison of capabilities, limitations, and applicability in similar contexts.

## CONCLUSIONS

This study represents a first exploration of the accuracy of ChatGPT-4 in designing individual weekly training plans for elite swimmers during a “specific” periodisation phase.

The mean scores given by coaches and athletes – 3.6 for distance swimmers and 3.7 for sprinters – indicate an overall neutral or slightly positive perception. The majority of coaches (65%) and a proportion of athletes (27.8%) felt that the system could be used in reallife training with minor modifications, while 34.8% of coaches and 47.2% of athletes would only accept it with significant changes. In addition, 25% of athletes rejected its use altogether.

Aerobic zones (A1) received the highest level of acceptance, suggesting that ChatGPT-4 is more reliable for designing low-intensity sessions. However, there was criticism of excessively long repetitions in A2 and sub-optimal structuring in B1 and B2. For zone C1, the programmes were rated positively in terms of intensity and structure, but the proposed frequency of sessions was considered excessive. The experts recommended limiting these sessions to once a week to avoid overloading and ensure optimal recovery.

The high intensity zones (C2 and C3) were well received in terms of intensity and structure, but the frequency was considered insufficient. Experts suggested increasing activation sessions in C3, particularly for sprinters, by replacing some A1 sessions.

In conclusion, ChatGPT-4 is a promising tool for generating basic training programmes, but cannot replace human expertise in areas that require personalisation and adaptation to the specific needs of athletes. Coach supervision remains essential to fine-tune technical details and ensure programme effectiveness in advanced stages of preparation.

Future research should go beyond the mere refinement of AI by exploring hybrid coaching models, where AI serves as a strategic support system while expert coaches make real-time adjustments. To ensure effective integration, it will be essential to develop systems capable of incorporating the principle of progressive overload and dynamically adapting training programmes based on performance data and expert feedback. The integration of machine learning algorithms could enable AI to progressively refine training prescriptions, improving their sustainability, personalisation and long-term effectiveness.

## References

[1] Ho B, Mayberry T, Nguyen KL, Dhulipala M, Pallipuram VK. ChatReview: A ChatGPT-enabled natural language processing framework to study domain-specific user reviews. Machine Learning with Applications. 2024; 15:100522. doi: 10.1016/j.mlwa.2023.100522.

[2] Chen S, Zhang Y, Yang Q. Multi-Task Learning in Natural Language Processing: An Overview. ACM Comput Surv. 2024; 56:1–32. doi: 10.1145/3663363.

[3] Hasani AM, Singh S, Zahergivar A, Ryan B, Nethala D, Bravomontenegro G, et al. Evaluating the performance of Generative Pre-trained Transformer-4 (GPT-4) in standardizing radiology reports. Eur Radiol. 2023; 34:3566–3574. doi: 10.1007/s00330-023-10384-x. 37938381

[4] Barlas T, Altinova AE, Akturk M, Toruner FB. Credibility of ChatGPT in the assessment of obesity in type 2 diabetes according to the guidelines. Int J Obes. 2024; 48:271–275. doi: 10.1038/s41366-023-01410-5. 37951982

[5] Ghosh S, Zhao X, Alim M, Brudno M, Bhat M. Artificial intelligence applied to ‘omics data in liver disease: towards a personalised approach for diagnosis, prognosis and treatment. Gut. 2024; gutjnl-2023-331740. doi: 10.1136/gutjnl-2023-331740. PMC11874365 39174307

[6] Uchikov P, Khalid U, Dedaj-Salad GH, Ghale D, Rajadurai H, Kraeva M, et al. Artificial Intelligence in Breast Cancer Diagnosis and Treatment: Advances in Imaging, Pathology, and Personalized Care. Life. 2024; 14:1451. doi: 10.3390/life14111451. 39598249 PMC11595975

[7] Dixit S, Kumar A, Srinivasan K, Vincent PMDR, Ramu Krishnan N. Advancing genome editing with artificial intelligence: opportunities, challenges, and future directions. Front Bioeng Biotechnol. 2024; 11:1335901. doi: 10.3389/fbioe.2023.1335901. 38260726 PMC10800897

[8] Saifi I, Bhat BA, Hamdani SS, Bhat UY, Lobato-Tapia CA, Mir MA, et al. Artificial intelligence and cheminformatics tools: a contribution to the drug development and chemical science. J Biomol Struct Dyn. 2024;42: 6523–6541. doi: 10.1080/07391102.2023.2234039. 37434311

[9] Puce L, Ceylan Hİ, Trompetto C, Cotellessa F, Schenone C, Marinelli L, et al. Optimizing athletic performance through advanced nutritionstrategies: can AI and digital platforms have a role in ultraendurance sports? Biol Sport. 2024; 41:305–313. doi: 10.5114/biolsport.2024.141063. 39416500 PMC11475005

[10] Naja F, Taktouk M, Matbouli D, Khaleel S, Maher A, Uzun B, et al. Artificial intelligence chatbots for the nutrition management of diabetes and the metabolic syndrome. Eur J Clin Nutr. 2024; 78:887–896. doi: 10.1038/s41430-024-01476-y. 39060542

[11] Javidan AP, Feridooni T, Gordon L, Crawford SA. Evaluating the progression of artificial intelligence and large language models in medicine through comparative analysis of ChatGPT-3.5 and ChatGPT-4 in generating vascular surgery recommendations. JVS-Vascular Insights. 2024; 2:100049. doi: 10.1016/j.jvsvi.2023.100049.

[12] Lozić E, Štular B. Fluent but Not Factual: A Comparative Analysis of ChatGPT and Other AI Chatbots’ Proficiency and Originality in Scientific Writing for Humanities. Future Internet. 2023; 15:336. doi: 10.3390/fi15100336.

[13] Solomon T, Laye M. Examining the sports nutrition knowledge of large language model (LLM) artificial intelligence (AI) chatbots. OSF Registries; 2024. doi: 10.17605/OSF.IO/ZCKYA. PMC12165421 40512755

[14] Xu Y, Liu Q, Pang J, Zeng C, Ma X, Li P, et al. Assessment of Personalized Exercise Prescriptions Issued by ChatGPT 4.0 and Intelligent Health Promotion Systems for Patients with Hypertension Comorbidities Based on the Transtheoretical Model: A Comparative Analysis. J Multidiscip Healthc. 2024; Volume 17:5063–5078. doi: 10.2147/JMDH.S477452. 39539514 PMC11559245

[15] Dergaa I, Ben Saad H, El Omri A, Glenn J, Clark C, Washif J, et al. Using artificial intelligence for exercise prescription in personalised health promotion: A critical evaluation of OpenAI’s GPT-4 model. Biol Sport. 2024; 41:221–241. doi: 10.5114/biolsport.2024.133661. 38524814 PMC10955739

[16] Zaleski AL, Berkowsky R, Craig KJT, Pescatello LS. Comprehensiveness, Accuracy, and Readability of Exercise Recommendations Provided by an AI-Based Chatbot: Mixed Methods Study. JMIR Med Educ. 2024; 10:e51308. doi: 10.2196/51308. 38206661 PMC10811574

[17] Erol E, Arıkan H. Does ChatGPT provide comprehensive and accurate information regarding the effects, types and programming of core exercises? Turk J Kinesiol. 2024; 10:178–182. doi: 10.31459/turkjkin.1516614.

[18] Masagca RC. The AI coach: A 5-week AI-generated calisthenics training program on health-related physical fitness components of untrained collegiate students. J Hum Sport Exerc. 2024; 20:39–56. doi: 10.55860/13v7e679.

[19] Washif J, Pagaduan J, James C, Dergaa I, Beaven C. Artificial intelligence in sport: Exploring the potential of usingChatGPT in resistance training prescription. Biol Sport. 2024; 41:209–220. doi: 10.5114/biolsport.2024.132987. 38524820 PMC10955742

[20] Düking P, Sperlich B, Voigt L, Hooren BV, Zanini M, Zinner C. ChatGPT Generated Training Plans for Runners are not Rated Optimal by Coaching Experts, but Increase in Quality with Additional Input Information. J Sports Sci Med. 2024; 56–72. doi: 10.52082/jssm.2024.56. 38455449 PMC10915606

[21] Havers T, Masur L, Isenmann E, Geisler S, Zinner C, Sperlich B et al. Reproducibility and quality of hypertrophy-related training plans generated by GPT-4 and Google Gemini as evaluated by coaching experts. Biol Sport. 2025; 42(2):289–329. doi: 10.5114/biolsport.2025.145911. 40182716 PMC11963122

[22] Staff HC, Solli GS, Osborne JO, Sandbakk Ø. Long-Term Development of Training Characteristics and Performance-Determining Factors in Elite/International and World-Class Endurance Athletes: A Scoping Review. Sports Med. 2023; 53:1595–1607. doi: 10.1007/s40279-023-01850-z. 37178349 PMC10356634

[23] Stöggl TL, Sperlich B. The training intensity distribution among well-trained and elite endurance athletes. Front Physiol. 2015; 6. doi: 10.3389/fphys.2015.00295. PMC4621419 26578968

[24] Zacca R, Azevedo R, Chainok P, Vilas-Boas JP, Castro FADS, Pyne DB, et al. Monitoring Age-Group Swimmers Over a Training Macrocycle: Energetics, Technique, and Anthropometrics. J Strength Cond Res. 2020;34(3):818–827. doi: 10.1519/JSC.0000000000002762. 30113917

[25] Liu X, Matjiur R, Sonchan W, Charoenwattana S, Chainok P, Gay A, et al. The Effect of Concurrent Resistance Training on Tethered Force, Lower Limbs Strength, Anaerobic Critical Velocity, and Swimming Performance: A Randomized Controlled Trial. Physiologia. 2024; 4:454–464. doi: 10.3390/physiologia4040031.

[26] Feijen S, Tate A, Kuppens K, Barry LA, Struyf F. Monitoring the swimmer’s training load: A narrative review of monitoring strategies applied in research. Scandinavian Med Sci Sports. 2020; 30:2037–2043. doi: 10.1111/sms.13798. 32767794

[27] Tate A, Turner GN, Knab SE, Jorgensen C, Strittmatter A, Michener LA. Risk Factors Associated With Shoulder Pain and Disability Across the Lifespan of Competitive Swimmers. J Athl Train. 2012 Mar-Apr;47(2):149–58. doi: 10.4085/1062-6050-47.2.149. 22488280 PMC3418126

[28] Pink MM, Tibone JE. The painful shoulder in the swimming athlete. Orthop Clin North Am. 2000;31(2):247–261. doi:10.1016/s0030-5898(05)70145-0. 10736394

[29] Olbrecht J, Partners FG. The Science of Winning: Planning, Periodizing and Optimizing Swim Training. F&G Partners, Partners in Sports; 2015.

[30] Puce L, Marinelli L, Pierantozzi E, et al. Training methods and analysis of races of a top level Paralympic swimming athlete. J Exerc Rehabil. 2018;14(4):612–620. doi:10.12965/jer.1836254.127. 30276182 PMC6165978

[31] Olbrecht J. Determining training intensity and content. In: Olbrecht J, ed. The Science of Winning: Planning, Periodizing and Optimizing Swim Training. Antwerp, Belgium: F & G Partners; 2007.

[32] Pollock S, Gaoua N, Johnston MJ, Cooke K, Girard O, Mileva KN. Training regimes and recovery monitoring practices of elite British swimmers. J Sports Sci Med. 2019; 18(3):577–85. 31427881 PMC6683628

[33] Sweetenham B, Atkinson J. Championship Swim Training. Champaign (IL): Human Kinetics; 2003.

[34] Rodríguez FA, Mader A. Energy systems in swimming. In: World Book of Swimming: From Science to Performance. New York, NY, USA: Nova Science Publishers, Inc; 2011. p. 225. doi: 10.13140/2.1.3260.5128.

[35] Rushall BS. About the specificity of physical conditioning in swimming: It is a lot more specific than commonly believed. 2014.

[36] Salo, D., Riewald, S.A. Complete Conditioning for Swimming. Human Kinetics; 2008.

[37] Rossetto C. Totalmente nuoto. Calzetti Mariucci Editore; 2022. Nuoto; 616 p. ISBN: 9788860286642.

[38] Fernandes RJ, Carvalho DD, Figueiredo P. Training zones in competitive swimming: a biophysical approach. Front Sports Act Living. 2024; 6:1363730. doi: 10.3389/fspor.2024.1363730. 38563019 PMC10982397

[39] Joshi A, Kale S, Chandel S, Pal D. Likert Scale: Explored and Explained. BJAST. 2015; 7:396–403. doi: 10.9734/BJAST/2015/14975.

[40] Moen F. The coach-athlete relationship and expectations. Int J Humanit Soc Sci. 2014; 4:29–40.

[41] Sandbakk Ø, Tønnessen E, Bucher Sandbakk S, Haugen T. Training Philosophy: What Is It, and What Are the Main Components? Int J Sports Physiol Perform. 2024; 20(1):1. doi: 10.1123/ijspp.2024-0473. 39637843

[42] Eccles JS, Harold RD. Gender differences in sport involvement: Applying the eccles’ expectancy-value model. J Appl Sport Psychol. 1991; 3:7–35. doi: 10.1080/10413209108406432.

[43] Gabbett TJ. The training—injury prevention paradox: should athletes be training smarter and harder? Br J Sports Med. 2016; 50:273–280. doi: 10.1136/bjsports-2015-095788. 26758673 PMC4789704

[44] Sein ML, Walton J, Linklater J, Appleyard R, Kirkbride B, Kuah D, et al. Shoulder pain in elite swimmers: primarily due to swim-volume-induced supraspinatus tendinopathy. Br J Sports Med. 2010; 44:105–113. doi: 10.1136/bjsm.2008.047282. 18463295

[45] Walker H, Gabbe B, Wajswelner H, Blanch P, Bennell K. Shoulder pain in swimmers: a 12-month prospective cohort study of incidence and risk factors. Phys Ther Sport. 2012;13(4):243–249. doi: 10.1016/j.ptsp.2012.01.001 23068900

